# The Regulatory Potential of Long Non-Coding RNAs in Bipolar Disorder

**DOI:** 10.3390/ijms27073099

**Published:** 2026-03-28

**Authors:** Siqi Li, Yuhan Fu, Zhenzhen Wang, Yan Zhang, Tao Sun, Nan Miao

**Affiliations:** Center for Precision Medicine, School of Medicine, Huaqiao University, Xiamen 361021, China; 23013071016@stu.hqu.edu.cn (S.L.); 24014071007@stu.hqu.edu.cn (Y.F.); 23014071016@stu.hqu.edu.cn (Z.W.); 25013071022@stu.ecnu.edu.cn (Y.Z.); taosun@hqu.edu.cn (T.S.)

**Keywords:** bipolar, lncRNAs, mood disorder, neural system

## Abstract

Bipolar disorder (BD) is characterized by mood swings between mania and depression, sharing overlapping symptomatic and genetic risk factors with other mood disorders. Long non-coding RNAs (lncRNAs) show specific spatiotemporal precision in distinct cell types in the human brain, and understanding the precise mechanisms of lncRNAs in mood switching in BD is fundamental to deciphering the key molecular networks underlying BD diagnosis and therapy. In this review, we summarize the classification of BD subtypes, the differences between BD and multiple mood disorders, and the functional potential of lncRNAs in BD. Future studies of these lncRNAs will facilitate the development of RNA-based diagnosis for BD.

## 1. Introduction

BD is characterized by mood swings between mania and depression [1,2]. Depressive episodes often resemble unipolar depression, with overlapping symptoms [3,4]. It includes three subtypes: BD-I, BD-II, and cyclothymic disorder (CD). BD-I features severe manic episodes, sometimes with psychosis, and shares genetic similarities with schizophrenia (SCZ) [5]. BD-II includes less intense hypomanic episodes and major depressive episodes without psychosis, resembling major depressive disorder (MDD) [6]. CD involves chronic mood fluctuations with hypomanic and depressive symptoms that do not fully meet hypomanic or major depression criteria [7].

lncRNAs, exceeding 200 nucleotides with limited protein-coding potential, operate through both *cis*- and *trans*-regulatory mechanisms [8,9,10]. Brain-specific lncRNAs regulate neuronal differentiation, synaptic plasticity, and neural circuit formation by modulating chromatin remodeling, alternative splicing, and competing endogenous RNA (ceRNA) networks [11]. There is substantial evidence linking the dysregulation of lncRNAs to psychiatric disorders. For instance, in schizophrenia (SCZ), reduced lncRNA *NEAT1* affects oligodendrocyte differentiation and myelination [12]. In depression, lncRNA *MIR155HG* exerts antidepressant effects via the *miR-155*/*BDNF* axis [13]. In autism spectrum disorder (ASD), the upregulation of *MSNP1AS* influences neurite outgrowth and neuronal differentiation via the RhoA, Rac1, and PI3K/Akt pathways [14]. Furthermore, dysregulated lncRNAs may also affect neural circuits and emotion in BD [15]. For example, *NR_028138.1* mediates the *miR-5196-5p–TNF* network in the peripheral blood [16], while *FOXD3-AS1*, *GAS5*, and *DGCR5* are involved in apoptosis [17,18] and *MEG3* and *RMST* are implicated in oxidative stress [19,20], highlighting their significant roles in BD.

In this review, based on neurobiological changes, genetic variants, and clinical treatment, we first summarize the classification of BD subtypes and the differences between BD and other psychiatric disorders. Secondly, we introduce the roles of neurotransmitters, neurotrophic factors, miRNAs, and circRNAs in bipolar disorder. We also summarize the mechanisms, databases, and methodological techniques relevant to lncRNAs, and their regulatory potential in the diagnosis and treatment of BD. Through this approach, we hope to deepen our understanding of the underlying neural pathways and pathogenic mechanisms of lncRNAs in BD, thereby advancing the development of more accurate diagnostic tools and effective therapeutic strategies.

## 2. Methods

### 2.1. Literature Search and Study Selection

#### 2.1.1. Inclusion Criteria

1. Examine therapeutic criteria, episode features, neurobiological changes, genetic variations, and treatments for three BD subtypes.
2. Compare clinical, demographic, and genetic differences among the three BD subtypes.
3. Analyze similarities and differences between bipolar disorder and other psychiatric disorders in terms of symptoms, neurobiology, genetics, and treatments.
4. Study neurotransmitters, neurotrophic factors, miRNAs, and circRNAs in bipolar disorder.
5. Detail mechanisms, databases, sequencing, and experimental methods in lncRNA research.
6. Focus on lncRNA studies related to BD in blood, PBMCs, and human cell lines.
7. Explore brain-specific lncRNAs in BD patients and animal models.
8. Include studies with both in vitro and in vivo components only if in vitro data are separately extractable.
9. Include studies on lncRNAs (*AP1AR-DT*, *Malat1*, and *Meg3*) in BD diagnosis as the main focus.
10. Consider articles published in English up to 1 February 2026.

#### 2.1.2. Exclusion Criteria

1. Exclude studies not using miRNA or circRNA in interventions.
2. Exclude some studies on clinical treatment of BD and other psychiatric disorders.
3. Exclude simplified studies on lncRNA molecular mechanisms and methodologies.
4. Exclude non-peer-reviewed sources like conference abstracts, editorials, dissertations, or gray literature.
5. Exclude studies not published in English.
6. Exclude studies lacking relevant control groups or proper experimental design.
7. Exclude studies published after 1 February 2026.

### 2.2. Data Extraction and Synthesis of Evidence

The extracted elements were the authors/year, cell lines, methodology, outcomes, and results and supplementary data. Findings were included only with explicit numerical data due to inconsistent reporting. Multiple reviewers cross-checked tables narratively without subgrouping or statistical synthesis. Significant heterogeneity made quantitative meta-analysis unfeasible, so studies were categorized based on evidence supporting specific mechanisms.

### 2.3. Summary of Review Design and Methods of Studies Included

#### 2.3.1. Summary of Review Design

- Section 3 summarizes the similarities and differences among the three BD subtypes and compares BD with other psychiatric disorders.
- Section 4 introduces the regulatory roles of neurotransmitters, neurotrophic factors, miRNAs, and circRNAs in bipolar disorder.
- Section 5 provides a simplified overview of lncRNAs, including their mechanisms, related databases, and methods.
- Section 6 discusses the diagnostic and therapeutic potential of lncRNAs in BD patients, covering both in vitro and in vivo studies, and summarizes brain-specific lncRNAs and their regulatory roles in humans and animal models.
- Finally, we summarize recent advancements in the roles of lncRNAs in BD diagnosis and therapy in Section 7, along with their limitations concerning functional validation and associated metabolic and immune-inflammatory pathways in Section 8.

#### 2.3.2. Table and Figure Construction

- To classify the three subtypes of bipolar disorder (BD), in Section 3.1, we present therapeutic criteria, episode features, risk-associated genomic loci, and alterations in brain structure in Tables.
- To enhance the presentation of studies comparing BD with other psychiatric disorders in Section 3.2, we synthesize relevant descriptions and present Figures.
- In Section 4.2, we provide a clear overview of pertinent long non-coding RNA (lncRNA) databases by concisely presenting detailed information, including species, functions, and resources, in Tables.
- To provide a comprehensive understanding of the lncRNAs in BD diagnosis and therapy, we provide detailed information about lncRNAs in Tables, with up-/down-regulated lncRNAs in multiple samples shown in Figures.

#### 2.3.3. Reference Refinement

- Under the predefined criteria, full-text articles were evaluated for eligibility. Studies were excluded if they did not specifically report on lncRNAs in BD. In total, over 30 studies were removed in the initial submission, and 11 unrelated studies were eliminated during the two rounds of revisions. In this review, 233 valid references were included in the final version.

## 3. Basic Information About Bipolar Disorder

BD carries a higher suicide risk than unipolar depression [21] and shows significantly smaller volumes of the whole hippocampus [22,23]. Both BD-I and BD-II share manic and depressive episodes. Genetically, overlapping genes in two subtypes include *SHANK1*/*2*/*3* [24,25], *GABRB1*/*R1* [26,27], *ANK3* [24], *GRIA1* [28], and others [29,30,31,32]. Additionally, common susceptibility genes such as *SLC25A17* (rs5758064), *ZNF184* (rs67240003), *EMCN* (rs11936939), and *RPL10AP3* (rs6990255), along with lncRNA variants *RP11-6N13.1* (rs323509) and *CTC-447K7.1* (rs11167721), have been identified [33,34]. This underscores the need for research to find precise neurobiological markers that differentiate these subtypes.

In this part, we summarize the classification and characteristics of BD and highlight the differences between BD and other psychiatric disorders.

### 3.1. Classification

The classification of BD is complex and overlaps with that of MDD and SCZ, requiring precise diagnostic criteria. Here, we summarize the characteristics of BD subtypes in Table 1.

#### 3.1.1. BD-I

BD-I involves at least one manic episode with psychotic symptoms lasting over 7 weeks, often leading to social or occupational impairments and potential hospitalization (Table 1) [46,47,48,49].

**Neurobiological changes**

Neuroimaging reveals widespread gray matter reduction in areas of BD-I, including the frontal, temporal, and parietal lobes and hippocampal cortex [22]. Moreover, cortical thinning in the ventral PFC and temporal region have been identified in BD-I. In the hippocampus, volumes were found to be smaller across most subfields, including the hippocampal tail, subiculum, presubiculum, CA1-4, molecular layer, GC-ML-DG, and HATA [23]. Compared to SCZ patients, BD-I patients had significantly higher wave III (*p* = 0.0062) and wave VII (*p* = 0.0472) amplitudes in sudatory brainstem response patterns [39].

**Genetic variation**

BD-I is characterized by neuronal hyperexcitability, dysregulation of the reward system, hyperactivity of dopaminergic pathways, and mutations in genes such as *CACNA1C*, *ANK3* [24], *MAD1L1*, and *TMEM258* [25]. Variants of the *SYNE1* gene are associated with an increased BD risk through the dysfunction of Candidate Plasticity Gene 2 (*CPG2*) [36]. Additionally, psychosocial stress exacerbates vulnerability to BD-I [50].

**Clinical treatment**

The pharmacological management of BD-I frequently involves the use of mood stabilizers such as lithium, which are essential for both managing the disorder and reducing the risk of suicide; however, they necessitate vigilant monitoring due to potential toxicity [51,52,53]. For patients who cannot tolerate lithium, valproate is an alternative that provides rapid relief during agitated or mixed episodes, although it carries risks such as weight gain, hepatotoxicity, and polycystic ovary syndrome (PCOS) and is contraindicated during pregnancy [54,55]. Maintenance therapy typically employs lithium or lamotrigine, with patient adherence and regular follow-up appointments being critical components of effective management [56,57]. Electroconvulsive therapy (ECT) is effective for treatment-resistant mania [58]; short-term repetitive transcranial magnetic stimulation (rTMS) can improve cognitive function [59]; and bright light therapy (BLT) helps to alleviate depressive symptoms in BD [60].

#### 3.1.2. BD-II

BD-II is characterized by at least one hypomanic episode lasting over four days and a major depressive episode lasting over 2 weeks, without full mania [49,61,62]. BD-II is marked by emotion circuit disruptions, reduced serotonin function, over-active norepinephrine systems, and white matter abnormalities [52,53]. It is often misdiagnosed as MDD due to prominent depressive episodes and frequently co-occurs with anxiety disorders, substance use disorders, and physical conditions like cardiovascular diseases [63].

**Neurobiological changes**

Neuroimaging reveals local gray matter reduction such as in the left temporal pole in BD-II [22,42]. In the hippocampus in BD-II, there were significantly decreased CA1/4, GC-ML-DG, and molecular layer volumes when compared to HC [23]. In addition, BD-II showed an increased amygdala volume and neuron connectivity in the prefrontal cortex, basal ganglia, and insula, which are linked to widespread cognitive deficits [42,43].

**Genetic variation**

BD-II is linked to reduced levels in the promoter region of both *BDNF* on Chr. 11 and Prodynorphin (*PDYN*) on Chr.20 [41], as well as elevated mRNA levels of *SLIT3* [34].

**Clinical treatment**

Treatment guidelines for BD-II are often based on those for BD-I and depression, using mood stabilizers, atypical antipsychotics, and cognitive behavioral therapies [40,63]. Quetiapine is a first-line treatment due to its antidepressant, sedative, and anxiolytic effects [64,65]. Lamotrigine with a mood stabilizer is used to improve depressive symptoms [66], and selective serotonin reuptake inhibitors (SSRIs) with mood stabilizers help to avoid triggering mania [67]. Mild hypomanic symptoms can be managed with lithium or valproate, with short-term second-generation antipsychotics (SGAs) if needed [64]. For maintenance, lamotrigine prevents depressive relapse, while lithium and quetiapine address significant mood instability [64].

#### 3.1.3. CD

CD involves frequent and chronic mood swings between mild hypomanic and depressive episodes that do not fully meet the criteria for hypomanic or major depressive episodes [44,68]. It falls under bipolar spectrum disorders in the DSM-5 and ICD-11—the two principal classification frameworks for mental disorders [69]. CD often coexists with anxiety disorders, attention deficit and hyperactivity disorder (ADHD), and borderline personality disorder (BPD), complicating diagnosis and treatment [70,71]. Individuals with CD often show mood instability with cyclothymic and irritable traits [72,73], commonly seen in children and adolescents, with an equal distribution between males and females [69,74].

**Neurobiological changes**

Cyclothymic temperament is associated with reduced activation of the left lingual gyrus and bilateral cuneus, which is related to emotion-processing circuits [45].

**Clinical treatment**

Treatment often mirrors BD strategies, with low-dose lithium [74,75] and mood stabilizers like lamotrigine [76], although the latter has limited efficacy. Cognitive behavioral therapy (CBT) and well-being therapy (WBT) are beneficial for improving emotion regulation and circadian rhythm stability [69].

#### 3.1.4. Differences in Three Subtypes

**Clinical and demographic variation in BD subtypes**

Recent studies show that BD-I affects males and females equally, while BD-II is more common in females (55–65% of cases) due to factors like more depressive episodes, higher help-seeking behavior, and hormonal factors [77]. BD-II impairs daily functioning and increases suicide risks, with females attempting suicide more frequently [46,63,78]. CD, although not fully meeting the diagnostic criteria, can impair social or work functioning and increase the risk of developing BD-I or BD-II [7,74].

**Genetic regulation and variation in BD subtypes**

In BD-I, increased *NT-3* and *NT-4*/*5* [79,80], along with decreased Glu/Gln and Glu/GABA ratios in the anterior cingulate cortex (ACC), have been observed [81,82]. BD-I is also associated with *miR-206*, *BDNF*, *CACNA1C*, *ANK3*, *MAD1L1*, and *TMEM258* [34,37,38]. In contrast, BD-II is linked to low *BDNF* and *PDYN* levels [41] and high *SLIT3* [34] mRNA levels. In the serum of BD-II patients, *miR-7-5p*, *-23b-3p*, *-142-3p*, *-221-5p*, and *-370-3p* were upregulated [83]. However, the significance of these genes or miRNAs in CD are still unclear.

Above all, based on neuroimaging and transcriptional levels, the precise classification of BD subtypes will enable a better understanding for diagnosis and personalized treatment.

### 3.2. Differences Between Bipolar Disorder and Other Psychiatric Disorders

BD shares clinical features with various psychiatric disorders, like BD-I, SCZ, BD-II, and MDD, often resulting in misdiagnosis, especially in the presence of emotional instability or psychotic symptoms [84,85]. Here, we outline the distinctions between BD and these disorders.

#### 3.2.1. BD-I vs. SCZ

**Figure 1:**
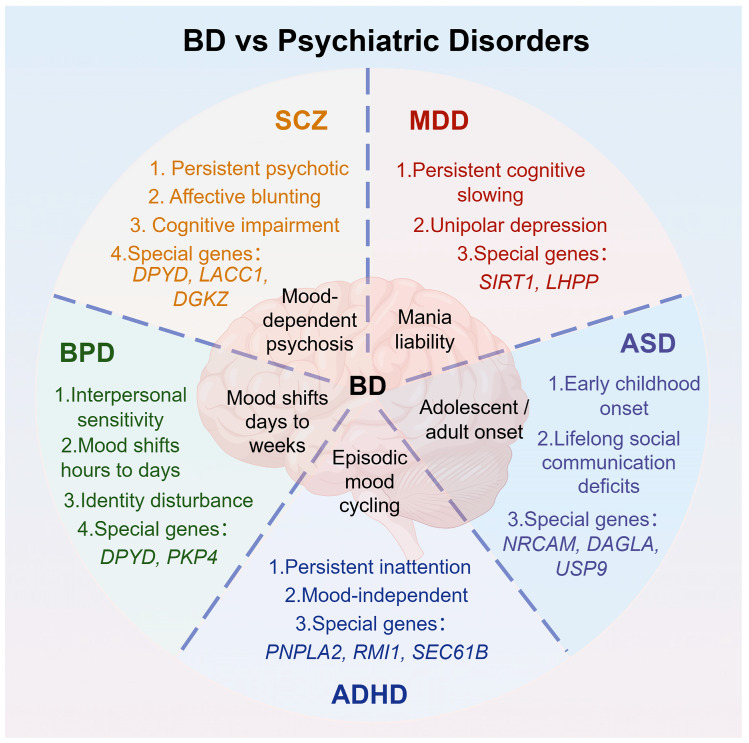
The differences between BD and other disorders.

BD-I and SCZ reside within a shared neurodevelopmental disease spectrum, both showing psychotic symptoms such as hallucinations and delusions but differing in timing and mood congruence [86,87] (Figure 1). Genetically, cross-disorder GWAS identified a schizophrenia–bipolar genetic factor, with risk loci mainly enriched in excitatory neurons in the hippocampal CA1/3 regions [87], including *SHANK3* [24] and *GRIA1* [28].

**Symptoms**

In BD, these symptoms align with mood episodes, while, in SCZ, they are persistent and detached from reality [88]. Cognitively, BD-I patients generally function well during stable periods, with slight impairments, whereas SCZ patients experience widespread, enduring cognitive deficits [88,89].

**Neurobiological changes**

Voxel-based morphometry shows that SCZ involves significant gray matter loss in the prefrontal cortex, temporal lobe, hippocampus, and cingulate cortex, but BD shows more localized changes, primary in the right thalamus and left insula, related to emotional regulation [90].

**Genetic variation**

Gene variants like *CACNA1C* (rs1006737) and *ANK3* (rs10994336) are linked to BD-I [37], and *DPYD*, *LACC1*, and *DGKZ* are associated with SCZ [91]. In addition, *GABRB1* (rs7680321) and *GABRR1* (rs9451173), both subunits of the GABAA receptor, selectively increase the risk for schizoaffective bipolar disorder (SABP) [26,27].

**Treatment strategies**

Treatment for BD often involves mood stabilizers and atypical antipsychotics, whereas that of SCZ relies on antipsychotics, which help with psychotic symptoms but have limited effects on cognitive and social functioning [92].

#### 3.2.2. BD-II vs. MDD

Approximately 40–60% of BD-II cases are first misdiagnosed as MDD due to shared symptoms such as low mood, anhedonia, fatigue, and reduced concentration [62]. Genetically, susceptibility genes are overlapping in these conditions, such as rs1006737 in *CACNA1C* and *RBKS* on Chr.2p [29,30].

**Symptoms**

BD-II includes hypomanic or mixed episodes, while MDD consists of only depressive episodes [93] (Figure 1). Cognitively, BD-II typically maintains preserved function during stable periods, with mild deficits, whereas MDD often involves ongoing attention and processing speed issues during acute episodes [93,94].

**Neurobiological changes**

Neuroimaging indicates that BD-II individuals often have increased connectivity in the prefrontal cortex, basal ganglia, and insula, with notable interhemispheric frontal connectivity [43]. In contrast, MDD patients show increased connectivity in the left frontal cortex, insula, and medial temporal regions, lacking the interhemispheric frontal connectivity seen in BD [43].

**Genetic variation**

BD has unique variants associated with mania, like *ADCY2* (rs13166360) [31], while MDD shows distinct risk loci including *SIRT1* (rs12415800) and *LHPP* (rs35936514) [95].

**Treatment strategies**

MDD is treated with antidepressants and psychotherapy, while BD often requires antipsychotics, with the cautious use of antidepressants to avoid inducing mania [96].

#### 3.2.3. BD vs. BPD

BD and BPD share traits like emotional instability, impulsivity, and interpersonal issues but differ in mood swing duration and triggers [97] (Figure 1).

**Symptoms**

BPD mood shifts are brief and sensitive to interpersonal events, while BD episodes last longer and follow a clear pattern, often independent of external stressors [97,98]. Cognitively, BD has mild, episodic deficits, while BPD shows ongoing issues in attention and emotion regulation [97]. During depressive phases, BPD shows more circadian rhythm disruption and self-harm, while BD is more associated with suicide attempts [99,100]. During stable periods, unlike BPD patients, who struggle with self-image, BD patients maintain a stable self-concept [101].

**Genetic variation**

Genetically, BD is linked to heritable risk variants, including rs10994336 in *ANK3*, rs12576775 in *ODZ4*, and rs1006737 in *CACNA1C* [32], whereas BPD is more connected to early trauma and personality development [102] and has unique risk genes like *DPYD* and *PKP4* [103]. *CACNA1C* is implicated in both disorders, indicating partial genetic overlap [32].

**Treatment strategies**

BD treatment focuses on medication, while BPD treatment emphasizes psychotherapy to enhance emotional control and reduce impulsivity [104].

#### 3.2.4. BD vs. ADHD

BD and ADHD share similar symptoms like attentional deficits, impulsivity, and emotional irritability in childhood, complicating diagnosis [105]. They also share comorbidity and genetic factors, as evidenced by GWAS findings of common risk loci like rs323509 near *RP11-6N13.1*, rs11167721 in *CTC-447K7.1*, and rs11936939 in *EMCN* [33,106].

**Symptoms**

ADHD is a persistent neurodevelopmental disorder lacking the mood cycles of BD, where attentional issues occur during manic episodes [107].

**Treatment strategies**

ADHD treatment typically involves stimulant and non-stimulant medications and behavioral therapies [108].

#### 3.2.5. BD vs. ASD

BD and ASD both involve emotional dysregulation, repetitive behaviors, and social withdrawal [109,110]. Genetically, BD and ASD share risk genes *ANK3* and *SHANK1*/*2* [25].

**Symptoms**

BD typically begins in adolescence or early adulthood with mood dysregulation, while ASD is marked by early social–communicative impairments and repetitive behaviors from infancy [111]. Although ASD and BD-II can co-occur, the social deficits of ASD are stable and unrelated to mood changes [112] (Figure 1).

**Neurobiological changes**

MRI scans show that ASD children have a slightly thicker cortex in the rostral middle frontal gyrus, while BD shows a thinner cortex there [113].

**Genetic variation**

Several ASD-specific genes have been validated, including *NRCAM*, *DAGLA*, and *USP9* [25].

**Diagnostic approaches**

Diagnostic tools like the Mood Disorder Questionnaire (MDQ) and Hypomania Checklist-32 (HCL-32), along with clinical follow-up, prove to be valuable for ASD [114].

## 4. Roles of Neurotransmitters, Neurotrophic Factors, miRNAs, and circRNAs in Bipolar Disorder

BD’s pathophysiology is complex, with 60–85% heritability [115], and environmental factors are categorized according to multiple developmental stages [116,117]. In this part, we highlight the roles of neurotransmitters, neurotrophic factors, miRNAs, and circRNAs in BD.

### 4.1. Neurotransmitters

BD involves neurotransmitter dysregulation, particularly in the dopamine transporter (DAT), glutamate (Glu), and GABA, affecting synthesis, transport, and receptor expression [118].

**Glutamine**

During depressive episodes, glutamate plus glutamine (Glx) and glutamine (Gln) are increased in the anterior cingulate cortex (ACC) [81]. The Glu/Gln ratio in the ACC is lower in euthymic BD-I patients due to the enhanced GAD1-mediated conversion of Glu to GABA, and mood stabilizers and antipsychotics can alter glutamine levels [119]. Unlike MDD with reduced Glx, BD shows higher whole-brain Glx levels without medication [120]. A higher BMI is linked to increased hippocampal Glx in BD, potentially due to increased pyruvate carboxylase activity [121,122]. Treatments like cytidine supplementation have reduced cerebral Glx levels and improved depressive symptoms in BD [123]. Studies have confirmed that *Malat1* downregulation drives neuronal hyperexcitability via enhanced glutamate-mediated calcium signaling, as confirmed in neuropathic pain rat spinal cord samples and cultured neurons [124].

**GABA**

In BD patients, decreased GABAergic transmission in the brain affects cognitive symptoms [82,125]. During the euthymic state, an elevated GABA/creatine ratio in the ACC and parieto-occipital cortex was seen [126], while a lower Glu/GABA ratio was observed in the dorsal anterior cingulate cortex in BD-I [82]. Gene variations in *MKLN1* (rs114034759) are linked to BD early onset via reduced expression, and *GADL1* (rs17026688 and rs17026651) is correlated with the lithium response through splicing-related mechanisms [127,128]. In the cerebella of BD patients, protein levels of *GAD65*/*67* and GABAB receptor subunits *GABBR1* (Chr. 6p21.3) and *GABBR2* (Chr. 5q34) are reduced [129,130]. The long-term use of mood stabilizers in BD enhances GABAergic neurotransmission and GABA receptor activity in the frontal cortex and hippocampus but reduces hypothalamic activity [131]. Valproate reduces high serum GABA levels in manic patients, while depressed patients typically have lower levels [132,133]. The lncRNA *17A*, within an intron of *GPR51*, alters GABA_B2 receptor splicing and increases amyloid-β secretion, affecting intracellular signaling and being associated with Alzheimer’s disease [134].

**DAT**

Mood swings in BD are linked to the DAT [135], with lower DAT levels in the striatum during manic phases, while its levels rise in euthymic states [136,137]. Additionally, rs27072 in the 3′-UTR of *SLC6A3* has been shown to reduce the dopamine transport capacity [138]. Due to the roles of DAT in BD, its agonists, like aripiprazole and cariprazine, can be used to treat manic and mixed episodes by reducing excessive dopaminergic signaling [139,140]. In addition, lithium reduces dopaminergic activity by increasing dopamine turnover and preventing dopamine receptor upregulation [141]. In SCZ, lncRNA *NONHSAT089447* is abnormally expressed in PBMCs, and, in neuroblastoma cells, decreased *NONHSAT089447* downregulated the dopamine receptor levels via *DRD3* and *DRD5* [142].

### 4.2. Neurotrophic Factors

During depressive episodes in BD, the levels of NT-3 and NT-4/5 were found to be higher in BD-I than in BD-II and healthy controls (HCs) but decreased during manic or euthymic states under lithium treatment for 6 weeks [79,80]. NT-3 gene polymorphisms (rs6489630 and rs11063714) are linked to visuospatial components [143], suggesting a role in stress regulation via the HPA axis [144]. Elevated NT-4/5 levels in manic and depressive episodes in BD are possibly affected by decreased BDNF-mediated corticostriatal transmission, leading to mood instability and cognition [145]. BDNF mRNA was downregulated in iPSCs in BD, upregulated in NSCs, and markedly reduced in the postmortem prefrontal cortex, while *BDNF-AS* remained unchanged [146]. Treatments like lithium and valproic acid can enhance BDNF by improving symptoms [147], and BDNF *Val66Met* (rs6265) variant carriers increase emotional and cognitive risks in BD [148].

During manic episodes in BD, low GDNF levels are positively linked to lithium response outcomes with high-definition transcranial direct current stimulation (HD-tDCS) [149]. Combining psychoeducation with medication in BD adults raises GDNF levels and reduces depressive symptoms more effectively [150]. Additionally, NGF levels are positively associated with the risk of MDD-to-BD transition, reflecting compensatory neurotrophic activation related to synaptic remodeling and front-limbic excitatory–inhibitory imbalance [151]. Elevated NGF levels have been identified in manic BD patients [152], highlighting its diagnosis potential via inflammatory pathways [153].

Modulating neurotrophic pathways could serve as a potential targeted therapy to improve neuroplasticity and resilience in BD.

### 4.3. miRNAs

miRNAs are critical post-transcriptional regulators and participate in biological processes related to BD, making them potential biomarkers for its diagnosis and treatment [154].

The transcriptomic profiling of blood, plasma, and neural tissues has revealed numerous dysregulated miRNAs in BD. For example, *miR-29a-3p* and *-125a-3p* [155] and *miR-140-3p* and *-21-3p* [156] were upregulated in BD whole blood, while increased *miR-132*, *-134*, *-152*, *-607*, *-633*, and *-652* and decreased *miR-15b* and *-155* were found in the plasma of BD patients [157]. During acute mania in BD, *hsa-miR-25-3p*, *-451a*, and *-144-3p* were upregulated, while *hsa-miR-4454*/*7975*, *-873-3p*, *-548a1*, *-598-3p*, *-4443*, *-551a*, and *-6721-5p* were downregulated; these were related to metabolism and neurodevelopment genes in plasma [158]. In the serum of BD-II patients, *miR-7-5p*, *-23b-3p*, *-142-3p*, *-221-5p*, and *-370-3p* were upregulated [83]. Moreover, elevated *let-7e-5p* and *miR-125a-5p* have been found in both BD and MDD plasma [159]. Compared to MDD, *miR-499*, *-708*, and *-1908* were decreased during depressive episodes in the plasma of female BD patients [160], and *miR-708-5p* was increased in the peripheral blood mononuclear cells (PBMCs) of male BD individuals [161]. *miR-15b*, *-132*, and *-652* were increased in high-risk BD individuals [162], and *miR-206* (rs16882131) and BDNF (rs6265) polymorphisms increased the BD-I risk [38], further suggesting that miRNAs contribute to BD susceptibility.

Pharmacological treatments alter miRNA expression based on the tissue and context. In manic BD, increased *miR-134* in the plasma and *miR-320a* and *-155-3p* in the whole blood were observed with lithium or valproate treatment [163,164]. In patient-derived lymphoblastoid cell lines (LCLs), lithium administered for 4-16 days upregulated *miR-34a*, *-152*, *-155*, and *-221* [165]. Conversely, in the rat hippocampus, *miR-34a*, *-221*, *let-7b*, *-7c*, *miR-128a*, *-24a*, and *-30c* were downregulated, whereas *miR-144* was upregulated [166]. Similarly, in cultured rat cerebellar granule cells, lithium and valproate downregulated *miR-34a* and *-495*, but *miR-182*, *-147*, and *-222* were upregulated [167].

Above all, miRNAs connect genetic risks, immune dysregulation, and synaptic dysfunction in BD, serving as valuable biomarkers and pharmacodynamic markers for mood stabilizers.

### 4.4. circRNAs

circRNAs are abundant in the brain and change dynamically during development, with their dysregulation linked to BD [168]. For example, *circHomer1a*, derived from *HOMER1*, is markedly downregulated in the prefrontal and orbitofrontal cortex and iPSC-derived neurons of BD and SCZ patients, and its knockdown in the mouse orbitofrontal cortex disrupted cognitive flexibility and synaptic gene-splicing profiles [169]. Similarly, *cNEBL* and *cEPHA3* were reported to be upregulated in the postmortem medial frontal gyrus in BD [170]. In addition, *circNCF1* and *circLINC00969* were downregulated in the PBMCs of SZ and BD patients [171]. In the peripheral blood of BD patients, 50 upregulated and 44 downregulated circRNAs were found, including upregulation on Chr.6 and 7 and downregulation on Chr.5 and 19 [172]. In postmortem ACC, circRNAs *circCCNT2*, *circCLOCK*, and *circRERE* were upregulated, while *circUBR5*, *circCYFIP2*, and *circLRBA* were downregulated [173]. Notably, *circCCNT2* was downregulated by lithium in vitro [173]; it may interact with RNA-binding proteins and *miR-877-5p* [174].

Collectively, circRNAs likely modulate neuronal transcriptomes via miRNA and RBP interactions, shaping BD-related cognitive and affective circuits.

## 5. Basic Information About lncRNAs

Since lncRNAs have limited protein-coding potential and weaken species conservation, the precise regulatory mechanisms of lncRNAs in BD are still unexplored. In this part, we first introduce the regulatory mechanisms of lncRNAs and then we highlight the databases on lncRNA annotation, prediction, and their related networks. Finally, we summarize the sequencing and experimental methods applied in lncRNA study.

### 5.1. Mechanisms of lncRNAs

lncRNAs influence gene expression through various mechanisms by interacting with DNA, RNA, and proteins [175]. Epigenetically, they can recruit chromatin-modifying complexes like PRC2 and HDACs to change the chromatin structure and regulate gene transcription [176]. At the transcriptional level, lncRNAs can affect nearby or distant genes by modulating enhancer activity or forming chromatin loops through cis or trans mechanisms [177]. Post-transcriptionally, they act as ceRNAs by binding to miRNAs, thus preventing miRNAs from inhibiting target mRNAs [178]. Additionally, lncRNAs interact with transcription factors or RNA-binding proteins (RBPs), serving as scaffolds or decoys to influence protein complexes [179]. They also impact alternative splicing, mRNA stability, and translation efficiency [179]. Some lncRNAs even encode micropeptides involved in cellular signaling and physiological regulation [180]. These mechanisms highlight the complex regulatory roles of lncRNAs in BD.

### 5.2. Databases for lncRNA Analysis

Central to advancing this field is the development of comprehensive databases that facilitate the quantification and functional validation of lncRNAs. Here, we outline the public and specific databases for lncRNA research in Table 2.

Public databases, including Ensembl, which encompasses 300 species, offer annotations, potential functions, and mapping for lncRNA analysis [181]. Due to the lack of conserved sequences and structural features, resources such as LNCipedia and LncBook provide multi-omics integration, disease associations, and regulatory networks specifically for humans [182,183]. For the analysis of miRNA target prediction and RBP interactions, databases like LncRBase [185], LncRNASNP [188], and offer comprehensive resources for researchers investigating lncRNA functions.

Specialized resources have been developed, such as lncSEA for ceRNA network and tissue specificity [184]; LncRNADisease [186] for disease association; and RNALocate [138] for subcellular localization. In conclusion, the expansion and refinement of lncRNA databases are instrumental in facilitating the functional validation and quantification of these non-coding elements.

### 5.3. Sequencing Methods for lncRNA Study

Multiple bulk-transcriptomic methods can be used to quantify lncRNAs, with RNA-seq being the primary tool for profiling their expression, while microarrays provide a cost-effective alternative for known lncRNAs [189].

Single-cell RNA sequencing (scRNA-seq) and scStereo RNA-seq offer precise spatiotemporal data on lncRNAs, which are often underrepresented in bulk RNA sequencing [190,191,192,193,194,195]. Recently, the 10 × Genomics technology has become popular for scRNA-seq due to its high throughput, although studies on lncRNAs using this method are limited [196,197]. Most scRNA-seq studies on lncRNAs have used chip- or plate-based technologies like the Fluidigm C1 and SMART-seq protocols [198,199,200]. The advent of computational frameworks like ELATUS has significantly improved the identification of functional lncRNAs from scRNA-seq data by enhancing concordance with ATAC-seq profiles in single-cell multi-omics datasets [201].

### 5.4. Experimental Validation in lncRNA Study

lncRNAs are distinctively characterized by their high tissue and cell type specificity compared to protein-coding genes, which is linked to their regulatory functions. Similarly to protein-coding genes, quantitative reverse transcription PCR (qRT-PCR) is utilized for the sensitive validation of selected lncRNAs [202]. Moreover, in situ hybridization (ISH) and single-molecule fluorescence in situ hybridization (smFISH) techniques are utilized to ascertain the spatial localization of lncRNAs [203,204]. Furthermore, approaches such as gene silencing, overexpression, and various co-expression analyses are instrumental in elucidating lncRNA regulatory networks [205,206,207,208]. Nonetheless, challenges such as the low expression levels of lncRNAs and the limited accuracy of their annotation have significantly impeded their application in such studies.

Above all, the integration of sequencing-based approaches has further advanced our comprehension of lncRNA structures and their interactions.

## 6. Diagnostic and Therapeutic Potential of lncRNAs in Bipolar Disorder

**Figure 2:**
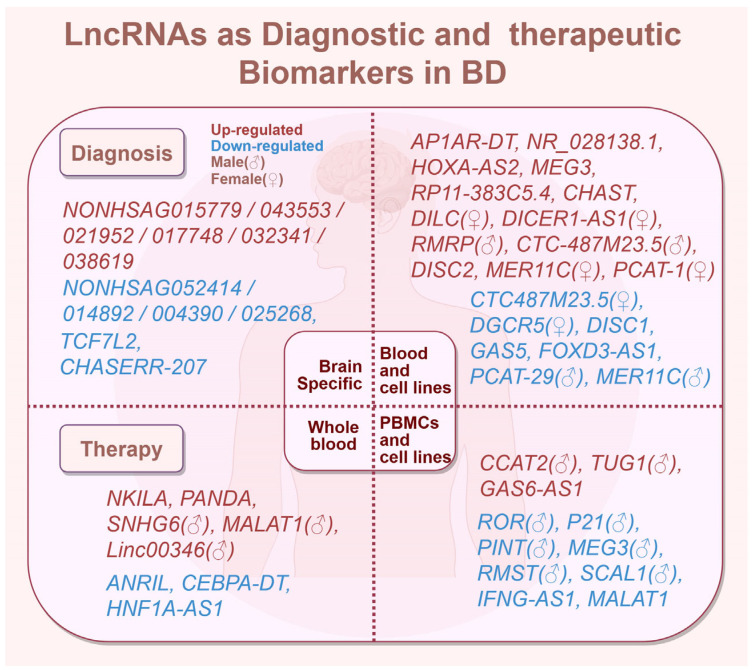
Diagnostic and therapeutic lncRNAs in BD.

lncRNAs, which are known to regulate gene expression and participate in various cellular processes, have been implicated in the pathophysiology of BD [209]. For example, decreases in *MEG3*, *RMST*, and *SCAL1* have been observed in BD, suggesting their potential roles as peripheral biomarkers for the disorder [20] (Figure 2). In this part, we discuss the diagnostic and therapeutic potential of lncRNAs in bipolar disorder.

### 6.1. Diagnostic Potential of lncRNAs in BD

Utilizing RNA sequencing, meta-analysis, and quantitative real-time PCR across various tissues from individuals with BD and HCs, abnormally expressed lncRNAs have been confirmed [16,210,211,212,213,214,215]. We systematically summarize the fold changes (log_2_FC), significance levels (*p*-values), and areas under the receiver operating characteristic curve (AUC) of potential diagnostic lncRNAs by comparing BD patients with HCs in Table 3.

#### 6.1.1. Abnormally Expressed lncRNAs in Blood and PBMCs in BD

Several studies of lncRNAs have shown that their expression levels are significantly elevated in BD patients compared to controls, but their exact roles remain unclear due to low density and weak conservation. Here, we summarize the abnormally expressed lncRNAs in the blood and PBMCs of BD patients (Table 3).

**Blood**

*AP1AR-DT* was upregulated in the peripheral blood of monozygotic twins with BD (HC = 4 twin pairs, BD = 5 twin pairs); it suppressed *NEGR1* by interfering with NRF1 binding or transcriptional complex assembly in neuronal nuclei in vitro [211]. Additionally, *NR_028138.1* (HC = 116, BD = 130) and *MEG3* (HC = 50, BD-I = 50) were found to be upregulated in the peripheral blood of BD patients [16,223]. Similarly, in the whole blood of BD-I patients and HCs (*n* = 50), *HOXA-AS2* and *MEG3* were notably upregulated [216].

**PBMCs**

In the PBMCs of BD-I patients (*n* = 50), *MEG3* is reported to be decreased fivefold [20]. By integrating prefrontal cortex datasets with PBMC and whole-blood validation, upregulated *RP11-383C5.4* was identified as a potential BD biomarker [213]. During a depressive episode, *CHAST* was upregulated in both sexes, while *DILC* and *DICER1-AS1* were specifically increased in female PBMCs [8]. Additionally, sex-biased expression has been identified in the PBMCs of BD-I patients (*n* = 50); for example, *RMRP* and *CTC-487M23.5* were upregulated in males, while *CTC-487M23.5* and *DGCR5* were downregulated in females [18].

Moreover, in the PBMCs of BD-I patients, increased *DISC2* and decreased *DISC1* may be altered by oxidative stress through heightening neuronal vulnerability and disrupting Glu/NMDA receptor signaling [219]. Conversely, *GAS5* and *FOXD3-AS1* were significantly downregulated in BD-I [17]. Another study of PBMCs in BD-I and HCs (*n* = 50) suggested that *PCAT-29* and *MER11C* were downregulated in males, while *MER11C* and *PCAT-1* were upregulated in females [15]. Conversely, *PCAT-29*, *MER11C*, and *PCAT-1* were upregulated in MDD patients [224], underlining their diagnostic specificity.

Despite these promising results, the application of these lncRNAs is still in the preclinical stage and they require further validation, standardized testing, and evaluation before clinical application.

#### 6.1.2. Brain-Specific lncRNAs in BD

The brain generates numerous non-coding RNAs that are crucial for neural cell development and synaptic plasticity. Their dysregulation can result in neuropsychiatric disorders like BD. Here, we review the genetic variations, dysregulation, and functions of brain-specific lncRNAs in BD.

**Genetic variation**

Genetic variation highlights the role of lncRNA-related networks in BD susceptibility. In postmortem human cortical samples, *TCF7L2* expression was downregulated, and, in hiPSC-derived astrocytes, the BD–BMI risk SNP (rs12772424) reduced *TCF7L2* expression [222]; this regulated BD risk genes *NCAN*, *TENM4*, and *NFIA* [212]. In addition, *AP1AR-DT* is near the PGC3 risk locus rs13106460, where genetic variation may affect lncRNA-driven transcription, potentially raising mood disorder susceptibility [225].

**Abnormally expressed lncRNAs in BD patients**

In the medial frontal gyrus in four BD patients and four HCs, *NONHSAG015779*/*043553*/*021952*/*017748*/*032341*/*038619* were upregulated, and *NONHSAG052414*/*014892*/*004390*/*025268* were downregulated; these are involved in angiogenesis, vascular development, and H3-K4 histone demethylation [170].

In the RNA-seq of postmortem striatal tissue in BD and SCZ patients (HC = 36, BD = 8, Table 3), *CHASERR-207* was significantly downregulated. It is regulated by over 20 miRNAs and targets the pro-apoptotic gene *PAWR*, which is associated with cognitive impairment [210].

**Functional validation of brain-specific lncRNAs in animal models**

While brain-specific lncRNAs have been confirmed in patients, their precise mechanisms of action are still unexplored. In animal models, several lncRNAs are associated with bipolar-like behaviors.

***AP1AR-DT*** overexpression in the mouse medial prefrontal cortex (mPFC) led to a reduction in the total spine density and the spontaneous excitatory postsynaptic current (sEPSC) frequency and depressive and anxiety-like behaviors [211]. Conversely, overexpressing *Negr1* in the mPFC neurons of these mice alleviated these behaviors and restored excitatory synaptic transmission. This study highlights *AP1AR-DT*’s role in modulating depressive and anxiety-like behaviors in BD.

***Malat1*** is highly expressed in brain tissue, and several lines of evidence suggest that it is involved in synapse generation and other neurophysiological pathways [226]. In addition, *Malat1* knockout mice exhibited the upregulation of neighboring gene *Neat* [227], which may be involved in high-phosphate (HP)-induced anxiety behavior [228].

***Meg3*** is weakly expressed in embryonic stem cells but upregulated during neuronal maturation, especially in glutamatergic neurons [229,230]. Clinically, in the hippocampus, increased *Meg3* with reduced promoter methylation is linked to extreme emotional behaviors [31]; it may disrupt neuronal balance by regulating *miR-7* and *miR-219* and the PI3K/AKT pathway [216,225,231].

### 6.2. Drug Response Potential of lncRNAs in BD

The exploration of drug responses in BD has increasingly focused on the roles of lncRNAs, which modulate pathways such as apoptosis, inflammation, and synaptic function. In this section, we summarize lncRNAs involved in BD drug responses across blood, PBMC, and neuronal models (Figure 2).

**Whole blood**

In the whole blood of BD-I patients (n = 50) treated with carbamazepine, the NF-κB-related *ANRIL*, *CEBPA-DT*, and *HNF1A-AS1* were downregulated, while apoptosis-related *NKILA* was upregulated in both sexes [217]. In addition, male BD patients (n = 50) exhibited the significant upregulation of vitamin D receptor-associated *SNHG6*, *MALAT1*, and *Linc00346* [218].

**PBMCs**

Under carbamazepine treatment, the apoptosis-related *CCAT2* and *TUG1* were upregulated in male BD-I patients (n = 50) under chronic oxidative and inflammatory apoptotic priming [19], whereas oxidative stress-related *lncRNA-p21*, *ROR*, and *PINT* were linked to p53/PTEN signaling [220], and *MEG3*, *RMST*, and *SCAL1* were downregulated in male BD patients (n = 50) [20].

In PBMCs from BD and SCZ patients (HC = 32, BD = 30), valproate and risperidone downregulated the inflammatory-related lncRNA *IFNG-AS1* by decreasing *IFNG* and *IL1B* via histone methylation [215]. *MALAT1* was further decreased after valproate and lithium treatment; it functions as a ceRNA involved in synaptic and developmental regulation [221].

**Cell lines**

Valproate-treated NT2-N neuronal-like cells exhibited upregulation of the hub *GAS6-AS1*, implicating PI3K/AKT-dependent synaptic vesicle trafficking and neurotransmitter release in the drug response [214].

## 7. Conclusions

This review summarizes recent advances in lncRNAs in BD, with a focus on their tissue specificity and treatment responsiveness. By outlining BD subtypes, comparing BD with related psychiatric disorders, and discussing neurotransmitter-related pathways, we highlight the potential molecular mechanisms underlying dysregulated lncRNAs in BD. Despite progress in studying lncRNAs in blood and brain tissues, challenges remain due to low expression levels, strong tissue specificity, and limited functional validation. The future integration of knockout models and single-cell sequencing may clarify how lncRNAs regulate neurotransmitter signaling and emotional state transitions, facilitating clinical translation. Overall, lncRNAs represent promising biomarkers and potential therapeutic targets in BD, offering new avenues for personalized diagnosis, intervention, early detection, and improved patient outcomes.

## 8. Limitations

This review symmetrically reviewed the current literature on lncRNAs in BD, highlighting their involvement in transcriptomic changes in brain or blood tissues. The low density and conservation of lncRNAs present challenges in elucidating the precise mechanisms of lncRNA–gene pairs or ceRNA networks in differentiating BD subtypes. The integration of scRNA-seq and scStereo-seq holds promise in enhancing our understanding of lncRNA–gene interactions. However, the inherent complexity of human BD samples renders scRNA-seq profiling particularly challenging. Furthermore, limitations associated with 10× Genomics impede the comprehensive understanding of the lncRNA *cis*-regulatory network, including transcription factors and chromatin accessibility. To investigate specific lncRNA–gene pairs in *cis*, it is essential to integrate scRNA-seq with assays for ATAC-seq within a single-cell multi-omics framework.

In addition, current studies ignore metabolic and immune–inflammatory factors affecting gene expression, potentially confounding lncRNA changes. BD patients often have metabolic syndrome and high pro-inflammatory cytokines, which may alter lncRNA expression, resulting in secondary disease signals. Future research should combine metabolic and immune data with multi-omics to better understand lncRNAs’ regulatory roles in BD.

## Figures and Tables

**Table 1:** The characteristics of BD subtypes.

Type	Therapeutic Criteria	Episode Features	Risk Loci	Brain Structural Changes
BD-I	Over 1 manic episode > 7 days	Elevated/irritable mood, grandiosity, hyperactivity, risk-taking [35]	*SYNE1* [36] *CACNA1C* [37] *ANK3* [37] *MAD1L1* [34] *TMEM258* [34] *miR-206* [38] *BDNF* (rs6265) [38]	Widespread gray matter reduction and cortical thinning in ventral PFC and temporal regions [22]. Hippocampal volume reduction [23]. Increased sudatory brainstem response patterns [39].
BD-II	Over 1 hypomanic episode > 4 days; Over 1 depressive episode > 1–2 weeks	Hypomanic episode: elevated mood, hyperactivity Depressive episode: low mood, anhedonia, sleep/appetite changes, suicidal ideation [40]	*BDNF* [41] *PDYN* [41] *SLIT3* [34]	Reduced gray matter in left temporal pole and limited cortical thinning in left orbitofrontal gyrus [22]. Hippocampal volume reduction [23]. Increased amygdala volume [42]. Increased connectivity in the prefrontal cortex, basal ganglia, and insula [43].
CD	At least 2 years chronic mood fluctuations	Recurrent subthreshold hypomanic and depressive symptoms, >50% duration, symptom-free intervals ≤ 2 months [44]	/	Reduced left lingual gyrus and bilateral cuneus [45].

**Table 2:** Summary of commonly used lncRNA databases.

Database	Species	Function	Source
Public			
Ensembl [181]	300 species	Annotation, function, and mapping	https://www.ensembl.org/index.html https://www.ensembl.org, accessed on 16 January 2026
LNCipedia [182]	Human	Annotation, encoding potential, and secondary structure	https://lncipedia.org, accessed on 16 January 2026
LncBook [183]	Human	Multi-omics integration, disease association, and regulatory network	https://ngdc.cncb.ac.cn/lncbook/, accessed on 16 January 2026
Specific			
lncSEA [184]	Human	Chromatin regulators, ceRNA mechanisms, and tissue specificity	http://bio.liclab.net/LncSEA/index.php, accessed on 16 January 2026
LncRBase [185]	Human, mouse, rat, chicken, zebrafish, Drosophila melanogaster, cow, and *C. elegans*	lncRNA–miRNA interaction, prediction, and validation	http://dibresources.jcbose.ac.in/zhumur/lncrbase2/, accessed on 16 January 2026
LncRNADisease [186]	Human	Annotation, disease associations	http://www.rnanut.net/lncrnadisease/, accessed on 16 January 2026
RNALocate [187]	242 species	Subcellular localization and functional analysis	http://www.rnalocate.org/, accessed on 16 January 2026
LncRNASNP [188]	Human, chimpanzee, pig, mouse, rat, chicken, zebrafish, fruit fly	SNP annotation in lncRNAs, miRNA binding sites, and secondary structure prediction	http://gong_lab.hzau.edu.cn/lncRNASNP3/, accessed on 16 January 2026

**Table 3:** The statistical features of BD.

lncRNA	Tissue	Participants	Log_2_FC	*p*-Value	ROC	Ref.
*AP1AR-DT*	Peripheral blood	HC twin pairs = 4, BD twin pairs = 5	+0.8329	0.0494	/	[211]
*NR_028138.1*	Whole blood	RNA-seq cohort: HC = 4, BD = 4; Clinical validation: HC = 116, BD = 130	+1.585	<0.01	0.923	[16]
*HOXA-AS2*	Whole blood	HC = 50, BD-I = 50	+1.57	0.003	0.70	[216]
*MEG3*			+1.47	0.015	0.71	
*ANRIL*	Whole blood	HC = 50, BD-I = 50	/	0.0011	0.68	[217]
*CEBPA-DT*			/	<0.0001	0.65	
*HNF1A-AS1*			/	<0.0001	0.86	
*NKILA*			/	0.0007	0.71	
*SNHG6*	Whole blood	HC = 50, BD = 50	/	<0.0001	0.94	[218]
*MALAT1*			/	<0.0001	0.95	
*Linc00346*			/	0.012	0.83	
*RP11-383C5.4*	PBMC	HC = 254, BD = 47	/	/	0.81	[213]
*CHAST*	PBMC	HC = 50, BD-I =50	+4.18	<0.0001	0.83	[8]
*RMRP*	PBMC	HC = 50, BD-I = 50	+4.13	<0.0001	0.80	[18]
*CTC-487M23.5*			+1.38	0.049	0.68	
*DISC1*	PBMC	HC = 50, BD-I = 50	−2.47	<0.0001	0.76	[219]
*DISC2*			+3.82	0.0015	0.68	
*GAS5*	PBMC	HC = 50, BD = 50	−5.1	<0.0001	0.90	[17]
*FOXD3-AS1*			−2.4	0.0028	0.84	
*PCAT-29*	PBMC	HC = 50, BD-I = 50	−5.30	<0.0001	0.76	[15]
*MER11C*			−3.49	0.0033	0.68	
*CCAT2*	PBMC	HC = 50, BD-I = 50	/	0.006	0.69	[19]
*TUG1*			/	<0.001	0.72	
*lncRNA-p21*	PBMC	HC = 50, BD = 50	−2.54	0.0055	0.66	[220]
*ROR*	PBMC	HC = 50, BD = 50	−4.26	0.0001	0.75	[20]
*PINT*			−3.34	0.0016	0.66	
*MEG3*			−2.25	0.011	0.63	
*RMST*			−2.46	0.001	0.70	
*SCAL1*			−2.12	0.022	0.61	
*IFNG-AS1*	PBMC	HC = 32, BD = 30	/	<0.0001	0.81	[215]
*MALAT1*	PBMC	HC = 50, BD-I =50	−2.39	<0.0001	0.80	[221]
*CHASERR-207*	Postmortem basal ganglia	HC = 36, BD = 8	−21.134	2.52 × 10^−6^	/	[210]
*TCF7L2*	hiPSC-derived astrocytes	HC = 1020, BD-I = 388	/	2.85 × 10^−8^	/	[222]
*GAS6-AS1*	NT2-N cells	24 cell samples	/	0.002	/	[214]

## Data Availability

No new data were created or analyzed in this study. Data sharing is not applicable to this article.
